# Analysis of wheat gene expression related to the oxidative stress response and signal transduction under short-term osmotic stress

**DOI:** 10.1038/s41598-019-39154-w

**Published:** 2019-02-26

**Authors:** Karolina Dudziak, Magdalena Zapalska, Andreas Börner, Hubert Szczerba, Krzysztof Kowalczyk, Michał Nowak

**Affiliations:** 1 Institute of Plant Genetics, Breeding and Biotechnology, Faculty of Agrobioengineering, University of Life Sciences in Lublin, 15 Akademicka St., 20-950 Lublin, Poland; 2 Leibniz Institute of Plant Genetics and Crop Plant Research (IPK), Corrensstrasse 3, D-06466 Stadt Seeland, Gatersleben Germany; 3 Department of Biotechnology, Microbiology and Human Nutrition, Faculty of Food Science and Biotechnology, University of Life Sciences in Lublin, 8 Skromna St., 20-704 Lublin, Poland

## Abstract

Water shortage is a major environmental stress that causes the generation of reactive oxygen species (ROS). The increase in ROS production induces molecular responses, which are key factors in determining the level of plant tolerance to stresses, including drought. The aim of this study was to determine the expression levels of genes encoding MAPKs (*MAPK3* and *MAPK6*), antioxidant enzymes (*CAT*, *APX* and *GPX*) and enzymes involved in proline biosynthesis (*P5CS* and *P5CR*) in *Triticum aestivum* L. seedlings in response to short-term drought conditions. A series of wheat intervarietal substitution lines (ISCSLs) obtained by the substitution of single chromosomes from a drought-sensitive cultivar into the genetic background of a drought-tolerant cultivar was used. This source material allowed the chromosomal localization of the genetic elements involved in the response to the analyzed stress factor (drought). The results indicated that the initial plant response to drought stress resulted notably in changes in the expression of *MAPK6* and *CAT* and both the *P5CS* and *P5CR* genes. Our results showed that the substitution of chromosomes 3B, 5A, 7B and 7D had the greatest impact on the expression level of all tested genes, which indicates that they contain genetic elements that have a significant function in controlling tolerance to water deficits in the wheat genome.

## Introduction

Cereal crops are the basis of agricultural production in most countries. *Triticum aestivum* L., as one of the most commonly cultivated cereals in the world (next to rice and maize), is particularly important^1^. Currently, wheat cultivation covers 220 million ha^2^. According to the FAO, 680 million tons of wheat are produced annually^3^.

Water deficits represent a major global abiotic stress that limit plant productivity by inhibiting plant growth and development. Drought induces ROS overproduction and leads to the disruption of membrane integrity and osmotic balance in plant cells. The consequence of these changes is a reduction in crop quality and quantity, which causes crop yield losses^4–6^.

The level of ROS significantly increases under drought conditions. In plants, the enzymatic antioxidant system, which involves many enzymes, such as superoxide dismutase (SOD), catalase (CAT), and peroxidases (POX) including ascorbate peroxidase (APX), glutathione peroxidase (GP) and guaiacol peroxidase (GPX), protects cells against toxic ROS^7–9^. However, many studies indicate that CAT, APX and GPX play the most important roles in ROS scavenging^7,8,10–14^. It has been shown that the expression patterns of *APX, CAT* and *SOD* in barley under drought conditions depend on the plant development stage and genotype^15^. The results of previous studies indicate that genes encoding antioxidant enzymes are frequently important in developing plants with enhanced drought tolerance. Increased tolerance to drought and salt stress has been observed in *Nicotiana tabacum* that overexpress the *APX* gene^16^. In transgenic rice, *OsMT1* gene overexpression indicates a higher level of CAT and APX activity and causes an increase in drought resistance^17^. Increased tolerance to abiotic stress factors (including drought) was found in an *Arabidopsis thaliana GP* overexpression line^18^.

The accumulation of osmolytes such as proline is another plant defense mechanism against stress conditions. Proline is synthesized in a two-step process catalyzed by ∆^1^-pyrroline-5-carboxylate synthetase (P5CS), followed by the reduction of P5C to proline by ∆^1^-pyrroline-5-carboxylate reductase (P5CR)^19^. Proline protective mechanisms during osmotic stress have been proposed to involve the stabilization of proteins and antioxidant enzymes, direct scavenging of ROS, balance of intracellular redox homeostasis, and cellular signaling promotion^20^. High *P5CS* expression was observed in rice treated with H_2_O_2_^21^. The upregulation of both *P5CS* and *P5CR* was found in *Brassica napus* under salt and ABA treatment conditions^22^. It has also been shown that the overexpression of the *P5CS* gene leads to an increase in proline accumulation and enhanced stress tolerance in tobacco^23,24^ and wheat^25^.

However, ROS are also involved in signal transduction in response to stress conditions^10,26,27^, and MAPK cascades are major signaling pathways^28^. This system consists of kinases (MAPK, MAP Kinases), kinase kinases (MAPKK, MAP Kinase Kinases) and kinase kinase kinases (MAPKKK, MAP Kinase Kinase Kinases)^28,29^. In the *Arabidopsis thaliana* genome, 80 MAPKKKs, 10 MAPKKs and 20 MAPKs have been identified^28,30–32^. The function of wheat MAPKs is under intensive study;^33–37^ however, there is still little data available. MAPK3 and MAPK6 are the best-described plant MAP kinases^32^, and their function is well established in many species. Most data on MAP kinases are based on the analysis of transcript levels. In wheat, changes in the expression patterns of *TaMAPK3* and *TaMAPK6* genes were found under drought conditions and salinity stress as well as during phosphor and nitrogen deficiency^35^ and *Mycosphaerella graminicola* infection^33^. Studies on rice indicate that *OsMAPK6* is involved in signal transduction during *Magnaporthe grisea* attack^38^, while *OsMAPK3* is involved in the response to cold stress^39^. There are several studies in maize concerning the activation of *ZmMAPK3* under cold, drought, salinity, the presence of heavy metal ions, UV and wounding^40^. The induction of *AtMAPK3* and *AtMAPK6* genes was observed in *A. thaliana* that was exposed to excess cadmium and copper^41^.

Many studies have focused on the development of varieties with increased drought resistance due to the importance of wheat in global cereal production and the worldwide problem of water deficits. The main purpose of such strategies is commonly the identification of genomic regions involved in the stress response^42,43^, although there is still no data concerning the initial plant reaction to water shortage. However, previous studies conducted with transcriptional or proteomic profiling indicate that the short-term plant response to stress factors can play important role in regulating gene expression and physiological responses, e.g., during hormone treatment^44^ or salt stress^45^. Yang *et al*.^44^. observed that transcriptional changes caused by short ABA treatment were much stronger than 9 days treatment and promote expression of genes encoding ethylene and JA signaling components.

The research carried out so far has shown that drought occurring during the early developmental stage determines the induction of signaling pathways as well as modification of plant growth and metabolic profile crucial for acclimation^46,47^. Moreover, the research carried out so far has shown that there is a correlation between traits related to drought response and increased yield for wheat^48^. It has been also shown, that drought stress occurs during the seedling stage can influence grain production^49,50^ and quality parameters^51^.

The main objective of this study was to examine wheat responses to short-term drought, as measured by the expression level of genes involved in signal transduction (MAPK3 and MAPK6), the activity of the antioxidant system and the proline biosynthesis in common wheat seedlings. Because of the fact that the majority of analyzed genes is encoded by more than one gene, localized on different chromosomes, we decided to focus on holistic analysis based on consensus coding sequences. Furthermore, using intervarietal substitution lines (ISCSLs), our purpose was to describe genome regions involved to the highest extent in response to drought including both: genes expression and its regulation.

## Results

### Expression of genes encoding enzymes involved in ROS signaling (MAPK3 and MAPK6)

**Figure 1:**
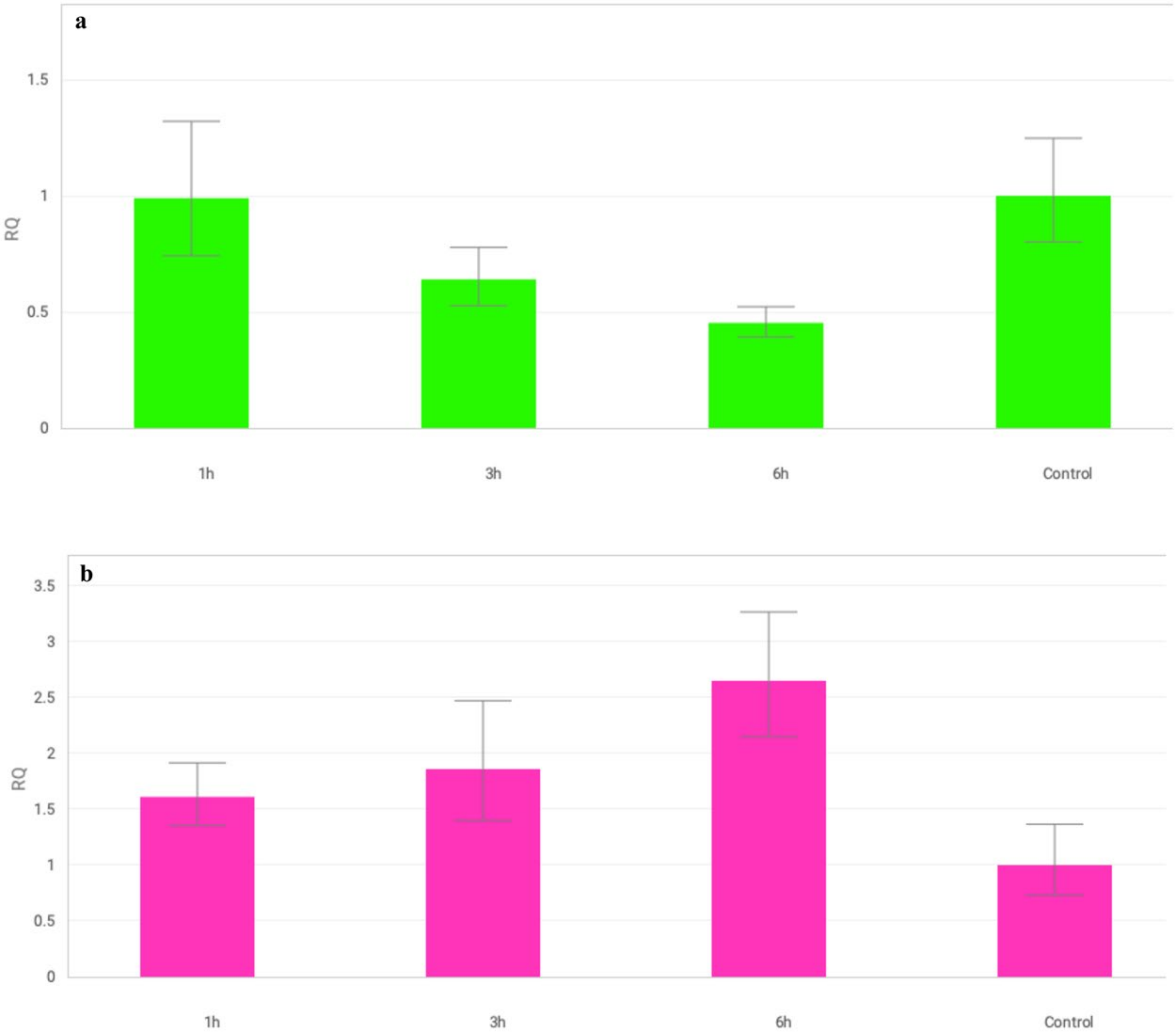
The pattern of *MAPK3* (**a**) and *MAPK6* (**b**) genes expression alteration in S29(JP) substitution lines after 1, 3 and 6 h of 10% PEG treatment. The average values obtained for all tested genotypes in particular time point are presented in comparison to non-exposed plants (Control). Bars represent standard deviation. All samples were analyzed in three full biological and three technical replications.

**Figure 2:**
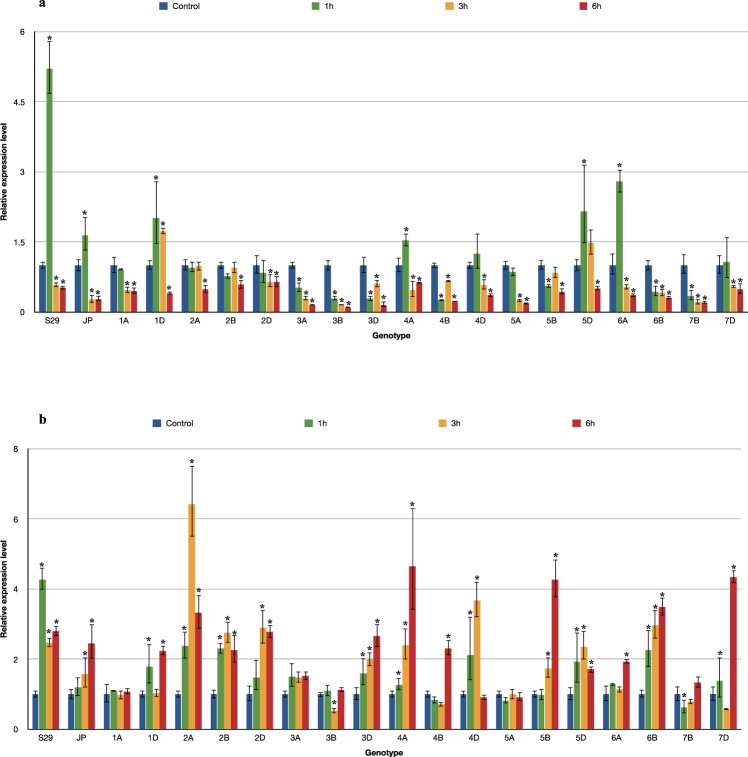
Changes in expression of *MAPK3* (**a**) and *MAPK6* (**b**) genes in S29(JP) substitution lines after 1, 3 and 6 h of 10% PEG treatment. Bars represent standard deviation. The expression level for non-exposed plants was used as calibrator (relative expression level = 1), *change significant at the 0.05 level. All samples were analyzed in three full biological and three technical replications.

The *MAPK3* and *MAPK6* genes showed two different patterns of expression in the tested *T. aestivum* L. seedlings exposed to drought (Fig. 1a,b). *TaMAPK3* transcript levels significantly decreased after 6 hours of exposure in most of the tested lines. However, different expression profiles were observed after 1 and 3 hours of stress for individual lines. Lines containing chromosomes of homoeologous groups 3 (3A, 3B, 3D), 4B, 6B and 7B showed a rapid response and immediate reduction in *MAPK3* expression after 1 hour of PEG treatment. The downregulation of *MAPK3* was noticed at subsequent time points (after 3 and 6 hours). Only 3D and 4B showed a transient increase in *MAPK3* expression after 3 hours. A significant induction of *MAPK3* was detected in the first hours of stress for the drought-tolerant cultivar S29 and lines 1D, 4A, 5D, 6A, followed by a rapid decrease in transcript levels. For the remaining forms, no *MAPK3* induction was observed in the first hours of plant exposure to drought, and then a significant decrease occurred. The biggest differences in the *MAPK3* expression pattern compared to the recipient were detected for lines 3A, 3B, 6B and 7B. The drought-sensitive cultivar JP showed a decrease in the transcript level after 3 hours. A similar expression pattern was recorded for 1A, 5A and 7D. *MAPK3* expression in lines 4D, 5B and 5D decreased only after 6 hours of water shortage. The results of the study indicated the weakest changes in *MAPK3* expression for lines containing chromosomes of homoeologous group 2 (2A, 2B, 2D) under the conditions tested (Fig. 2a).

Most ISCSLs showed an increase in the transcript level of *MAPK6* after 6 hours. However, different expression patterns were observed during stress treatment for individual lines. Rapid *MAPK6* induction already occurred after 1 hour in the drought-tolerant cultivar S29. Similar expression profiles were recorded for lines involving chromosomes of homoeologous group 2 (2A, 2B, 2D), 4D, 6B, 3D, 4A and 5D. A significant increase in *MAPK6* transcript levels was detected for those lines after 3 hours of 10% PEG exposure. Lines 3A, 3B, 5A, 6A and 7B showed the most significant changes in *MAPK6* expression compared to the recipient. The drought-sensitive cultivar JP exhibited a delayed response, and *MAPK6* expression was elevated significantly only after 3 and 6 hours. A similar delayed induction of *MAPK6* was noted for lines 4B, 5B and 7D. Most lines with the substitution of A genome chromosomes (1A, 3A, 5A) showed no significant changes in *MAPK6* expression throughout the 6 hours of stress (Fig. 2b).

### Expression of genes encoding antioxidant enzymes (CAT, APX and GPX)

**Figure 3:**
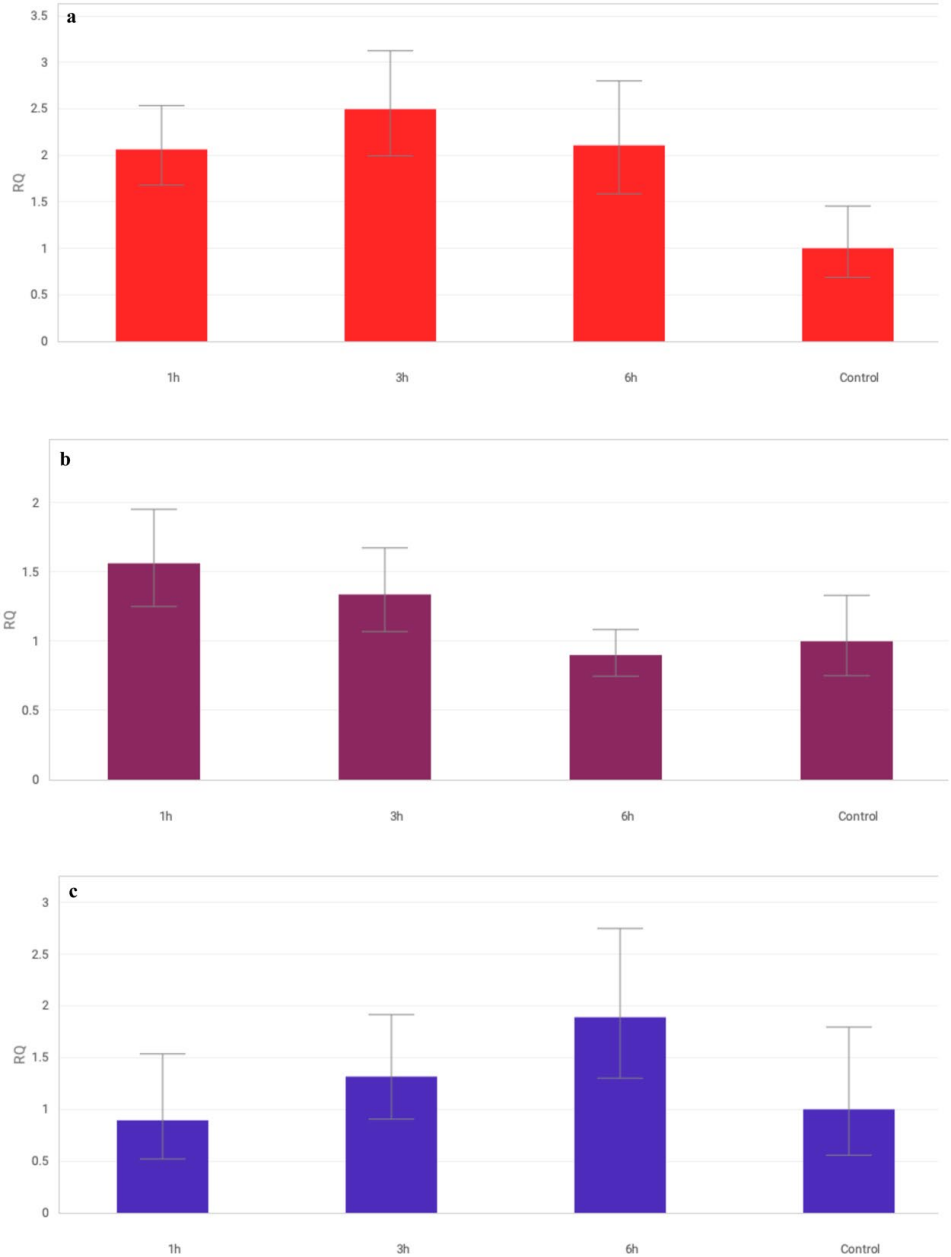
The pattern of *CAT* (**a**), *APX* (**b**), and *GPX* (**c**) genes expression alteration in S29(JP) substitution lines after 1, 3 and 6 h of 10% PEG treatment. The average values obtained for all tested genotypes in particular time point are presented in comparison to non-exposed plants (Control). Bars represent standard deviation. All samples were analyzed in three full biological and three technical replications.

**Figure 4:**
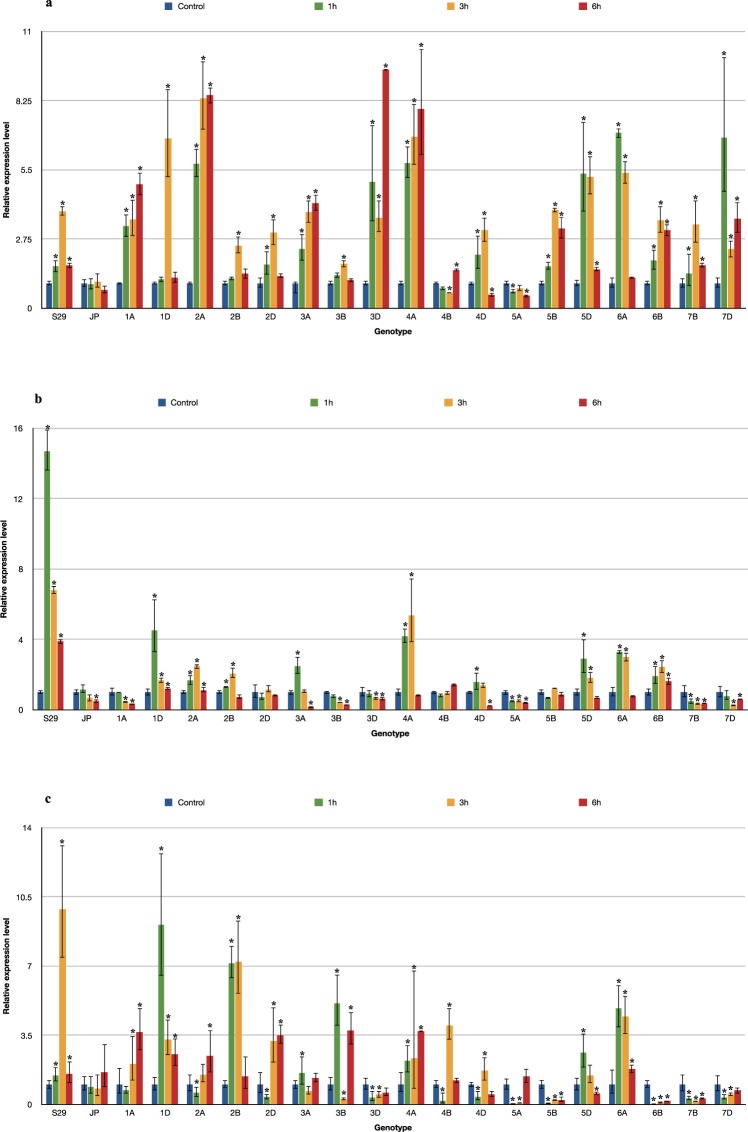
Changes in expression of *CAT* (**a**), *APX* (**b**), and *GPX* (**c**) genes in S29(JP) substitution lines after 1, 3 and 6 h of 10% PEG treatment. Bars represent standard deviation. The expression level for non-exposed plants was used as calibrator (relative expression level = 1), *change significant at the 0.05 level. All samples were analyzed in three full biological and three technical replications.

The overall pattern of *CAT* expression was a significant increase in transcript levels after 1 hour, which remained stable after 3 and 6 hours of stress (Fig. 3a). This expression trend was observed for lines with the substitution of A genome chromosomes (1A, 2A, 3A, 4A) and 3D, 5B and 6B forms. However, S29, ISCSLs with substitution of chromosomes from groups 2 (2B, 2D), 4 (4A, 4D), 7 (7B, 7D) and lines 1D, 5D and 6A initially showed an increase in *CAT* expression, followed by a decrease after 6 hours of drought. This expression pattern was characteristic for lines with the substitution of D genome chromosomes. The most significant alterations in *CAT* gene expression compared to the recipient (S29) were observed for lines 3B, 4B and 5A, that response was similar to the drought-sensitive cultivar JP (Fig. 4a).

The pattern for *APX* gene expression was characterized by an initial increase, followed by a decrease (Fig. 3b). However, elevated transcript levels were only observed for S29, 1D, 2B, 5D, 6B and lines with the substitution of A genome chromosomes (2A, 3A, 4A, 6A). Other lines showed lower transcript levels after 1 hour (1A, 4D), 3 hours (3B, 7D) or 6 hours (JP, 3A) exposure to stress. Lines with chromosomes belonging to homoeologous groups 3 (3B, 3D), 5 (5A, 5B), 7 (7B, 7D) and lines 1A, 2D, 4B showed the greatest changes in *APX* expression in response to drought stress compared to the recipient (S29). No significant changes in *APX* expression were observed for 2D, 3D, 4B or 5B under the conditions tested (Fig. 4b).

The analysis of the *GPX* gene indicated different expression patterns in the tested plants (Fig. 3c). S29 showed a significant increase in expression after 3 hours, followed by a decrease during prolonged drought (6 hours). A similar trend was observed for lines 1D, 2B, 4B, 5D and 6A. The substitution of chromosomes 3A, 3D, 5A, 5B, 6B, 7B and 7D had the greatest impact on *GPX* expression compared to the recipient. Continuous upregulation of *GPX* expression was recorded in lines with the substitution of A genome chromosomes (1A, 2A, 4A). Some of the tested lines exhibited a significant decrease in *GPX* expression during 10% PEG treatment: lines containing chromosomes of homoeologous groups 5 (5A, 5B) and 7 (7B, 7D) and line 6B. The transient downregulation of the *GPX* gene occurred in the first hours, followed by an increase in the transcript level for the majority of lines with the substitution of D genome chromosomes (2D, 3D, 4D, 7D). No significant changes of *GPX* expression were observed for the drought-sensitive cultivar JP (Fig. 4c).

### Expression of genes involved in proline biosynthesis (P5CS and P5CR)

**Figure 5:**
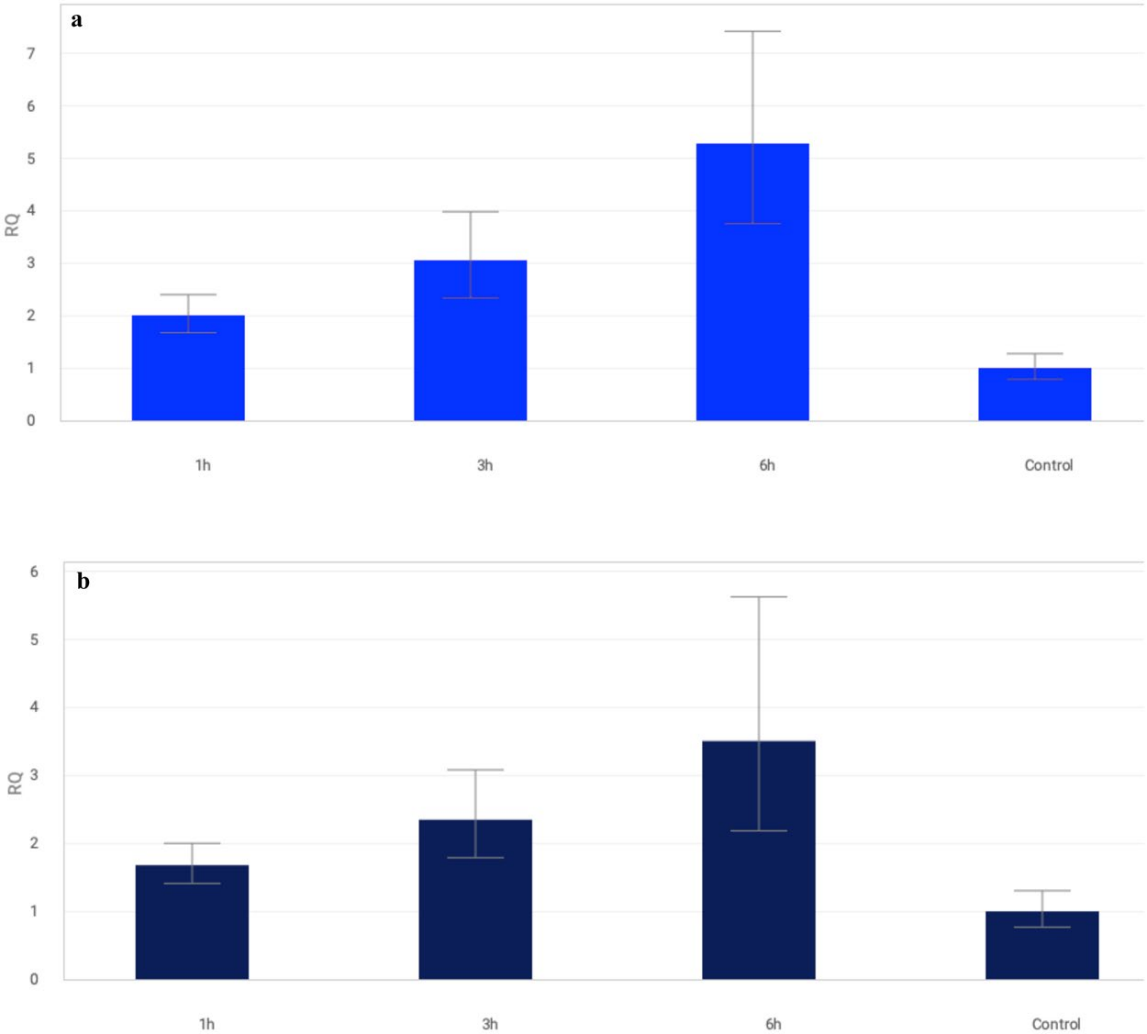
The pattern of *P5CS* (**a**) and *P5CR* (**b**) genes expression alteration in S29(JP) substitution lines after 1, 3 and 6 h of 10% PEG treatment. The average values obtained for all tested genotypes in particular time point are presented in comparison to non-exposed plants (Control). Bars represent standard deviation. All samples were analyzed in three full biological and three technical replications.

The results of the study indicated a significant increase in the expression of both genes: *P5CS* and *P5CR*. However, elevated transcript levels of the *P5CS* gene were observed immediately after 1 hour of stress, while a delayed induction (after 3 hours) was noticed for *P5CR* (Fig. 5a,b).

**Figure 6:**
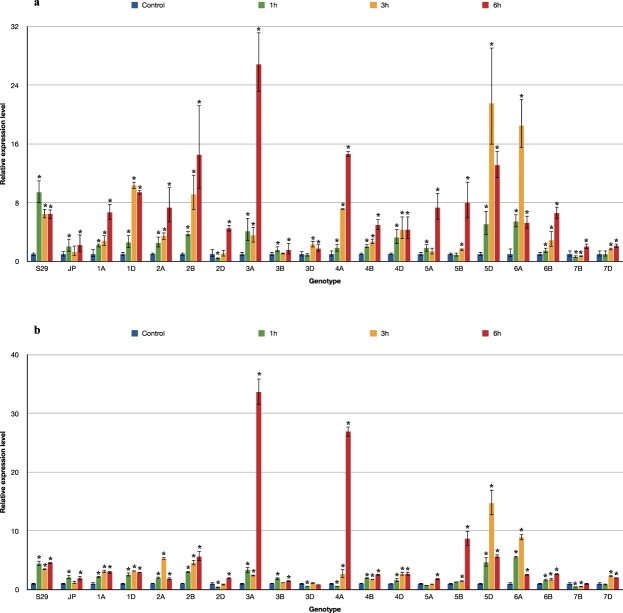
Changes in expression of *P5CS* (**a**) and *P5CR* (**b**) genes in S29(JP) substitution lines after 1, 3 and 6 h of 10% PEG treatment. Bars represent standard deviation. The expression level for non-exposed plants was used as calibrator (relative expression level = 1), *change significant at the 0.05 level. All samples were analyzed in three full biological and three technical replications.

The exposure of plants to drought resulted in a rapid response and increase in *P5CS* expression in most of the tested lines. S29, JP, 1D, 2B, 4B, 4D, 5D and lines with the substitution of A genome chromosomes (1A, 2A, 3A, 6A) already showed *P5CS* upregulation after 1 hour of stress. Moreover, the level of transcript for the drought-tolerant cultivar was significantly higher than that for the drought-sensitive one at each time point. A delayed response was observed for lines 3D, 4A and 6B (after 3 hours) and for 2D, 5A, 5B, 7B and 7D (after 6 hours of stress). Therefore, the results of the analysis showed that lines containing chromosomes from homoeologous groups 1, 2 and 4 exhibited a fast response to drought and continuous upregulation of *P5CS*, while a delayed reaction to stress was observed in lines containing chromosomes from homoeologous groups 5 and 7. Line 3B did not show any changes in P5CS gene expression. The transient downregulation of the *P5CS* gene was found in lines 2D and 7B after 1 hour (Fig. 6a).

The results showed similar trends in *P5CR* and *P5CS* regulation for the majority of the tested lines. A significant increase in *P5CR* expression was detected in S29 and JP after 1 hour of stress however, the upregulation of *P5CR* was higher in the drought-tolerant cultivar S29 than that in drought-sensitive JP. Similar observations were made for lines 1D, 2A, 2B, 4B, 5D and 6A. A delayed response to water stress, manifested as *P5CR* gene upregulation, was found in lines 4A, 4D, 7D (after 3 hours) and 2D, 5B, 6B (after 6 hours). A significant decrease in the *P5CR* transcript level was observed for 2D, 3D, 4A and 7B after 1 hour of drought. In comparison to the recipient (S29), lines 3B, 3D, 5A, 7B and 7D showed the highest changes in the *P5CS* and *P5CR* expression profile in response to drought (Fig. 6b).

## Discussion

Plant MAPK cascades have been intensively studied as a mechanism involved in the regulation of stress response. Many studies have indicated the important roles of MAPK3 and MAPK6 in plant cells^33,41,52,53^. However, the information available on MAPKs in *T. aestivum* is still limited^33,35–37^.

This study tested genes encoding two MAPKs (*MAPK3* and *MAPK6*), three antioxidant enzymes (*CAT, APX* and *GPX*) and two enzymes involved in proline biosynthesis (*P5CS* and *P5CR*). According to our data, the main pattern for the *MAPK6* gene was increased expression in drought-treated plants. *MAPK6* gene induction during water deficiency has also been reported in wheat^35^ and *A. thaliana*^32^. However, some of the tested lines did not show any changes in *MAPK6* expression (1A, 3A, 5A, 6A, 3B and 7B), which had also been demonstrated in wheat cultivar Chinese Spring using RNA-seq^36^. The downregulation of *MAPK3* gene expression was observed in all tested lines. The results suggest that kinase encoded by this gene may also function in signal transduction but as negative regulator A decrease in *MAPK3* expression was described for wheat under phosphorus deprivation^35^ and for *Cucumis sativus* L. under drought conditions^54^. However, our analysis of the *MAPK3* gene indicated different expression patterns for each line during 6 hours of PEG treatment. Based on previous data, we suggest that there is no unequivocal evidence for a specific *MAPK3* gene expression pattern under drought. Wen *et al*.^35^. reported no significant changes in *MAPK3* expression in wheat, while Zhan *et al*.^36^. observed an increase in the *TaMAPK3* transcript level. *MAPK3* expression upregulation was also described in *A. thaliana*^55,56^. Wang *et al*.^40^. reported a high level of *ZmMAPK3* expression after 1 hour of 10% PEG treatment and decreased expression after 6 hours. These authors suggest that there are common mechanisms underlying the abiotic stress signaling and convergent points that include the production of H_2_O_2_ generated by stress stimuli and the reliance on common signaling cascades. The versatile system also allows linking the H_2_O_2_ signal to the MAPK cascade and target genes. Identifying all cascade modules and broadening our knowledge about the regulation of all molecular mechanisms under stress conditions will provide further insight into the biological response of plants. Different profiles of *MAPK3* and *MAPK6* expression obtained in this study suggest that these kinases are regulated differently. Obtained results showed that *MAPK6* overexpression is one of the components of plant response to drought, whereas *MAPK3* do not play a role as a positive signal transduction regulator during this kind of stress. Moreover, a reverse reaction of *MAPK3* versus *MAPK6* expression in experimental conditions can indicate on the regulation mechanism based on feedback between these two kinases. Analysis based on ISCSLs revealed that chromosomes 3A, 3B, 6B, and 7B as well as 3A, 3B, 5A, 6A, and 7B contained genes involved in *MAPK3* and *MAPK6* expression regulation, respectively (Table 1). These results could provide a premise that chromosomes 3A, 3B and 7B contain genetic elements responsible for controlling of both analyzed kinases.

**Table 1:** Chromosomes involved to the highest extent in regulation of analyzed genes expression according to results obtained on the basis of examined ISCSLs set.

Gene	Chromosome
MAPK3	3A, 3B, 6B, 7B
MAPK6	3A, 3B, 5A, 6A, 7B
CAT	3B, 4B, 5A
APX	1A, 2D, 3B, 3D, 4B, 5A, 5B, 7B, 7D
GPX	3A, 5A, 5B, 6B, 7B, 7D
P5CS	3B, 3D, 5A, 7B, 7D
P5CR	3B, 3D, 5A, 7B, 7D

Water deficiency leads to ROS formation and the induction of genes that encode antioxidant enzymes. The involvement of those genes in promoting plant responses to unfavorable conditions has been well established in many studies^57–59^. It has been reported that the overexpression of genes encoding antioxidant enzymes causes higher tolerance to stress factors in *A. thaliana*^60^ and rice^16,61^. However, some data indicate that retaining stable gene expression can also confer drought tolerance in plants^62,63^.

In this study, the significant increase in *CAT* expression was observed in the majority of tested lines. *CAT* gene upregulation was also observed in wheat subjected to mild drought for 7 hours^64^, in *Macrotyloma uniflorum* after 78 hours^65^ and in barley after 2 days of drought^66^. High levels of *CAT* expression were observed in *Cleome gynandra* and *Cleome spinosa* during prolonged drought exposure (10 days)^67^. However, in this study, some of the tested lines showed a significant decrease in *CAT* expression after 6 hours of water stress. Previous studies have revealed variable responses to different durations and severities of drought stress in various plant species. A lower *CAT* transcript level was observed in wheat after 10 days^68^ or in barley after 9 and 16 days of stress^66^. Our results suggest that short-term drought increased *CAT* expression as a rapid plant response. However, prolonged exposure to drought could lead to the inhibition of expression and the reduction in transcript levels. PEG (10%) did not alter *CAT* expression in JP and lines 3B, 4B, and 5A, what can indicate the presence of encoding or regulatory elements on these chromosomes (Table 1). Similar observations were reported for *Poa pratensis*^57,62^ and *Koeleria macrantha*^53^. Moreover, in this work, we report similar expression patterns for *MAPK6* and *CAT* genes. Xing *et al*.^69^. suggested that *MAPK6*, as a component of a cascade involved in signal transduction, mediates H_2_O_2_ formation and *CAT* expression changes. Our results can support this hypothesis and indicate, that both enzymes are involved in the complex immediate reaction to osmotic stress in wheat.

Our analysis of the genes encoding two peroxidases showed that no constitutive expression pattern can be defined for all analyzed lines (especially for *GPX*). One of the potential factors responsible for this situation is fact that both of these enzymes are encoded by numerous sequences localized in different parts of the wheat genome. The trend of response to examined stress revealed enhancement of both genes expression, however, the time shift between them was observed. For *APX* the quick response was noticed, whereas for *GPX* a slower building up of the response was shown.

*APX* gene showed its expression downregulation in lines 3B, 5A, 7B and 7D. A reduction in *APX* transcript level was reported previously for *Arachis hypogaea* L^70^. and in roots of *P. pratensis* under drought treatment conditions^62^. No significant changes were found in the leaves of *P. pratensis*, which is consistent with our results obtained for lines 2D, 3D, 4B and 5B. The upregulation of the *APX* gene was observed in lines with the substitution of A genome chromosomes (2A, 3A, 4A, 6A), lines 2B, 5D, 6B and the drought-tolerant cultivar S29. Many previous studies have pointed to an increase in *APX* expression in different plant species under drought conditions, including *Pisum sativum* L.^71^, *K. macrantha*^63^, *P. pratensis*^57^ and *Hordeum vulgare* L.^66^. We observed a higher *APX* expression level in S29 compared than that in JP. This outcome has also been reported in drought-tolerant and -sensitive barley under water shortage conditions^66^. Some data indicate that *APX* gene overexpression enhances tolerance to drought in transgenic rice^53^ and tobacco^58^.

Our results of *GPX* expression analysis are consistent with those of previous studies performed on *K. macrantha*^63^ or *P. pratensis*^57,62^. *GPX* upregulation was detected in some of the tested lines. The induction of the *GPX* gene can be the result of higher H_2_O_2_ levels in plant cells or reduced enzyme activities^72^. An increase in GPX expression was also recorded for *K. macrantha*^63^, a drought-tolerant cultivar of *T. aestivum*^68^, and in wheat roots^72^. Our study showed a significantly elevated level of *GPX* expression in S29. This phenomenon has been previously reported for other tolerant *T. aestivym* cultivars after 10 days of drought^68^.

The results of our research based on ISCSLs suggest that genetic elements encoding and/or regulating the genetic activity of ascorbate peroxidase and guaiacol peroxidase may be present notably on chromosomes belonging to homoeologous groups 3, 5, and 7 (Table 1).

The current results for antioxidative system have demonstrated that catalase and ascorbate peroxidase, which can efficiently scavenge H_2_O_2_ and prevent its accumulation to toxic levels, are major antioxidant enzymes. However, these enzymes have different affinities for H_2_O_2_ and play different roles in scavenging. CAT does not need a reductant to scavenge H_2_O_2_, making it reducing power-free, whereas APX needs a reductant (ascorbate)^62,66,73^. According to our results, the *CAT* expression pattern was similar to *APX* in most of the tested lines (S29, JP, 1D, 2A, 2B, 3A, 4A, 4B, 5A, 5D, 6A, 6B). Transcript levels significantly increased in these forms after 1, 3 and/or 6 hours of stress. The results of the analysis indicated that this expression pattern was characteristic for lines with the substitution of A genome chromosomes and lines containing chromosomes of homoeologous groups 2, 4, 5 and 6. Similar regulation of *CAT* and *APX* expression could be explained by the fact that proteins encoded by these genes are both involved in the scavenging of H_2_O_2_ produced during oxidative stress, and their gene expression is likely regulated in the same way. Bian and Jiang^62^ suggested that *CAT* and *APX* may facilitate efficient H_2_O_2_ scavenging in leaf cells, although CAT and APX have different affinities for H_2_O_2_. No relationship was found between the expression of these two genes in the remaining lines. Different expression patterns have been described for tobacco plants exposed to drought, where an increase in *Cat3* and a decrease in *strAPX* expression occur^74^.

Proline is synthesized in a two-step process catalyzed by ∆^1^-pyrroline-5-carboxylate synthetase (P5CS), followed by the reduction of P5C to proline by ∆^1^-pyrroline-5-carboxylate reductase (P5CR)^19^. Both genes showed a significant increase in expression levels in the tested wheat substitution lines with 10% PEG treatment. *P5CS* and *P5CR* upregulation was also found in *K. macrantha* under water stress conditions^63^. Previous results show that increased expression of these genes may control plant responses and contribute to a higher level of RWC and proline accumulation or lead to a decrease in MDA content. The overexpression of genes involved in proline synthesis enhances drought tolerance in transgenic *Glycine max*^75^, *Petunia hybrida*^76^, *Cicer arietinum*^77^ and *T. aestivum*^25^. Our study demonstrated that the exposure of plants to 10% PEG resulted in a rapid response reflected by an immediate increase in *P5CS* and *P5CR* expression levels in the first hours of stress. This observation confirms the hypothesis, that the major mechanism underlying proline biosynthesis in response to drought is regulation at the transcriptional level. This mechanism has also been reported in other plant species under drought conditions. An increase in *P5CS* transcript levels was reported for *Oryza sativa* L. after 2 and 5 hours^78^, *Brassica napus* after 1, 2 and 6 hours^22^ and for *A. thaliana* after 5 and 12 hours of water stress^79^. *P5CS* gene upregulation has also been observed under osmotic stress conditions induced by NaCl in wheat^19^, moth bean (*Vigna aconifolia*)^80^, soybean^81^ and *A. thaliana*^82^. An increase in *P5CR* expression was described in *P. sativum*^83^ and *A. thaliana*^84^ after 6 and 12 hours of NaCl treatment, respectively.

Our results showed a higher level of *P5CS* expression in the drought-tolerant cultivar compared to that in the drought-sensitive one, which has also been found in rice^78^. In most of the tested lines, the *P5CS* gene showed an immediate induction after 1 hour of stress, while changes in *P5CR* were observed after 3 or 6 hours. These results suggest that the *P5CS* gene plays a key role in proline biosynthesis and the plant response to short-term drought. Similar expression patterns were reported for *A. thaliana*. Changes in *AtP5CS* gene expression were detected after 2 hours and in *AtP5CR* after 24 hours of osmotic stress^82^. These results can indicate that product of *P5CS* gene could induce *P5CR* expression as well. Analysis of the results obtained for each ISCSL indicates, that genes encoding enzymes associated with proline biosynthesis (*P5CS* and *P5CR*) or their regulators were located on chromosomes 3B, 3D, 5A, 7B, and 7D (Table 1). The location of these genes on 3B and 3D chromosome is supported by data from Phytozome (Table 2) however, the remaining genome regions indicated need further investigations. Moreover, lines with the substitution of the 3A and 4A chromosomes showed very strong upregulation of both proline biosynthesis genes expression after 6 hours of the experiment. This observation can indicate the presence of regulatory elements on these chromosomes.

**Table 2:** Phytozome transcripts annotated to selected antioxidant and proline biosynthesis enzymes, applied for consensus coding sequences construction.

Enzyme	EC	Phytozome transcripts
CAT	1.11.1.6	Traes_4DS_FA4454E51.1, Traes_7DL_44F6042FE.1, Traes_4BL_664A41517.1, Traes_7BL_7A3B8A199.1, Traes_4BL_825998751.1, Traes_4DL_4FC0D4B27.1, Traes_5AL_EEA9DF0FC.1,Traes_7AL_B42CCD94B.1, Traes_4BL_1852E26C9.1, Traes_6AS_7FB8F9A66.1, Traes_6DS_3522B8EF6.2, Traes_4DS_F0ABE9257.1, Traes_7AL_65B1F0872.1, Traes_7AL_F80BC8414.1
APX	1.11.1.11	Traes_4BL_19FA6DCAD.1, Traes_6AL_80FD46553.1, Traes_4DL_8CE055F15.1, Traes_5BL_C2D4F19B1.1, Traes_4AS_9EEABCE1C.1, Traes_2DL_59D310517.2, Traes_5AS_3AFCAA6AC.1, Traes_2AL_6FA87E31C.2, Traes_5DL_690A481C7.1, Traes_5BS_DA33416FB.1, Traes_6DL_2A99B8CDC.1, Traes_4BL_FBE8A057A.1, Traes_2BL_C8F030038.2, Traes_6BL_83DE6DC09.1, Traes_2AL_0EFC246E71.1, Traes_2AL_0EFC246E7.1, Traes_5BS_C8C312966.1
GPX	1.11.1.7	Traes_2DL_09743F9A9.1, Traes_7AS_090BA704D.1, Traes_3AS_634121F561.1, Traes_1BS_70E91236D.1, Traes_4AL_454E2A798.1, Traes_7AS_FE0E50F2E.1, Traes_1BS_C25C6D1AD.1, Traes_1BL_9D96A6922.1, Traes_7AS_0611176A1.1, Traes_7AL_AB00F8D69.1, Traes_6DS_EBBE8AE2A.2, Traes_1AL_1F3A0CD1F.1, Traes_4BL_4CDA94F8D.1, Traes_2BS_4FF4CEA9F.1, Traes_2DL_59588BD68.1, Traes_2BS_AABEC0F2F.1, Traes_2BS_19F05C27A.2, Traes_7DL_883CB0B5B.1, Traes_7DL_DB6471BF0.1, Traes_1DS_3D2F70A22.1
P5CS	2.7.2.11	Traes_3DL_3E215D878.2, Traes_3B_C4683D0FA.2, Traes_1DL_0BB66CF71.1, Traes_1BL_31105367B.1
P5CR	1.5.1.2	Traes_4AL_D98D91F71.1, Traes_2AL_9D35F6B8F.1, Traes_3B_1E5C683B5.1, Traes_3DL_EB6A17449.1

In conclusion, our study shed light on the molecular mechanisms of plant responses to short-term drought conditions. The first reaction of the tested wheat lines was characterized by changes in the transcript levels of *CAT*, a gene encoding an enzyme closely associated with H_2_O_2_ detoxification. Furthermore, stress induced the expression of genes involved in proline biosynthesis and the *MAPK6*-mediated signaling pathway. The results suggest that plant responses are controlled by differential gene expression regulation.

In the present study, we identified chromosomes associated with the initial wheat response to short-term stress using a set of S29 substitution lines (JP) with varying drought tolerance. The data indicated that the substitution of chromosomes 3B, 5A, 7B and 7D had the largest impact on the expression level of all tested genes and could play a critical function in controlling tolerance to water deficits in the wheat genome (Table 1). Moreover, we suggest that structural or regulatory genes involved in the first plant response to drought may be located on those chromosomes. Our results are consistent with previous data reported by several research groups^85–88^.

Further investigations should be performed, including gene expression analysis of other MAPKs and antioxidant enzymes as well as evaluation of their activity to gain new insight into drought tolerance in plants and to clarify the roles of these enzymes in the general pattern of the stress response. A better understanding of the mechanisms underlying drought tolerance will help to develop stress-tolerant genotypes. Moreover, mapping strategies should also be devised for the localization of genetic loci associated with wheat responses to drought.

## Methods

### Plant material and drought treatment

Wheat (*Triticum aestivum* L.) intervarietal substitution lines (ISCSLs), with ‘Saratovskaya 29’ (S29) as a recipient and ‘Janetzkis Probat’ (JP) as a donor, were used in the study. S29 is a drought-tolerant cultivar, and JP is a drought-sensitive one. The set of 18 ISCSLs kernels was provided by the Leibniz Institute of Plant Genetics and Crop Plant Research (IPK, Gatersleben, Germany).

Grains were sterilized with chlorine gas for 4 hours and transferred to Petri dishes containing filter paper soaked in distilled water. For germination induction, the grains were incubated at 4 °C for 48 hours and germinated for 2 days in the dark at 24 °C. Germinating seedlings were placed in plastic pots containing MS medium and maintained in a hydroponic culture in a growth chamber for 5 days under control conditions (light/dark regime of 16/8 h at 25 ± 3 °C, relative humidity of 50 ± 10%, light intensity during the daytime was 350 μmol m^−2^ s^−1^). For stress induction polyethylene glycol (PEG) addition to the medium was used. PEG cause osmotic stress occurrence, inducing plant water deficit similar to drought condition^89^. The main advantage of this experimental model is fact, that hydroponic culture provides a constantly reduced water potential, which is not possible in soil models^90^. Drought stress was induced after 5 days of acclimatization by applying 10% PEG-6000 dissolved in MS medium solution. To avoid hypoxia occurrence, the medium was aerated consistently. Wheat seedlings were collected after 1, 3 and 6 hours of stress treatment. Plants growing in MS medium without PEG were used as a control.

### Identification of analyzed genes transcripts and the detection system design

This study analyzed genes encoding antioxidant enzymes (CAT, APX, GPX), enzymes involved in proline biosynthesis (P5CS and P5CR) and two MAPKs (MAPK3 and MAPK6). For genes encoding antioxidant enzymes and enzymes involved in proline biosynthesis the Phytozome 12.1 (https://phytozome.jgi.doe.gov) database was used to identify full-length cDNA sequences within the wheat genome (*Triticum aestivum* v. 2.2) (Table 2)^91^. For identified sequences, multiple sequence alignment was performed using T-Coffee software with M-Coffee algorithm^92^ and the representative consensus sequence was built. For analyzed MAPKs the sufficient information about coding sequences was not present in Phytozome database. Because of that fact we decided to identify wheat ortologs of *A. thaliana MAPK3* and *MAPK6* genes in NCBI GenBank database. Based on the alignment results, the accessions AF079318 and AY173962 were selected for *MAPK3* and *MAPK6*, respectively. In order to confirm the homology of these sequences with respective *MAPK3* and *MAPK6* sequences from other selected plant species (*A. thaliana*, *Brachypodium distachyon*, *O. sativa* and *Zea mays*) an alignment was performed using BLAST tool (https://blast.ncbi.nlm.nih.gov) (Table 3). Obtained sequences were used as templates for detection system design. Gene-specific primers and probes were designed using PrimerBLAST software (https://www.ncbi.nlm.nih.gov/tools/primer-blast/)^93^ (Tables 4 and 5). For qPCR dual-labeled (6-FAM as a reporter, BHQ-1 as a quencher) TaqMan probes were applied.

**Table 3:** Alignment of the selected wheat *MAPK3* and *MAPK6* sequences with the corresponding sequences from the other species.

Species	Accession number	E-value
***MAPK3***		
*Arabidopsis thaliana*	NM_114433.3	1e-88
*Brachypodium distachyon*	NM_001279922.1	2e-39
*Oryza sativa*	DQ826422.1	0.0
*Zea mays*	EU130900.1	0.0
***MAPK6***		
*Arabidopsis thaliana*	NM_129941.4	0.0
*Brachypodium distachyon*	XM_003574199	4e-126
*Oryza sativa*	EU675863.1	0.0
*Zea mays*	EU965114.1	0.0

**Table 4:** Sequences of primers used in qPCR.

Primer	Primer (5′ → 3′) sequence
CAT-F	CACCTGGTGGAGAAGATCGC
CAT-R	TCACCTCGAAGAAGCCCTTG
GPX-F	GCGGTGACACCAACATCAAC
GPX-R	GTCCAGGTTCTCCAGGTTGG
APX-F	CAAGGCTCTGACCACCTCAG
APX-R	CATCTTCCCAGGGTGTGACC
P5CS-F	GATTCTCCGATGGTGCTCGT
P5CS-R	TTCAACACCCACAGGTCCAC
P5CR-F	TAAATGCCGTTGTTGCTGCC
P5CR-R	AGCAAAACTAACAATGGCTACCAG
MAPK3-F	CTTTAACCCGCTGCAGAGGA
MAPK3-R	GTCAAAGGAGAAGGGGTCCG
MAPK6-F	GAGGTCACCGCCAAGTACAA
MAPK6-R	CTTGTTGTCGAAGGCGTTGG
CJ705892-F	AACCACCGCATTTGCTGAAG
CJ705892-R	GACAGGGTGCCACCAACTAT

**Table 5:** Sequences of TaqMan probes used in qPCR.

Probe	Probe (5′ → 3′) sequence
CAT	[6-FAM]ACTTCGACCGCGAGCGCATC[BHQ-1]
GPX	[6-FAM]AGGCCAACTGCCCCCAGTCC[BHQ-1]
APX	[6-FAM]GCAGGTGTTTTCCACTCAGATGGGT[BHQ-1]
P5CS	[6-FAM]GGACTCGGTGCTGAGGTTGGC[BHQ-1]
P5CR	[6-FAM]ACAACAAGATGCCGAGAGCTCTCA[BHQ-1]
MAPK3	[6-FAM]AGAGGCGCTGGAGCACCCTT[BHQ-1]
MAPK6	[6-FAM]CCCCATCCTCCCCATCGGCA[BHQ-1]
CJ705892	[6-FAM]AGAGCCATTGTCTTGGCAGGCT[BHQ-1]

### Extraction of total RNA and qPCR (Real-Time PCR) analyses

Total RNA from wheat seedlings was extracted using TRIzol reagent (Invitrogen, USA) according to the manufacturer’s instructions. The quality and quantity of isolated RNA were evaluated by 2% agarose gel electrophoresis and spectrophotometrically using DeNovix DS-11. Total RNA (1.5 µg) was used to synthesize cDNAs using the iScript™ cDNA synthesis kit (Bio-Rad). Quantitative PCR was performed with TaqMan probes. PCRs were carried out in a total volume of 20 µl containing 80 ng of cDNA, 1× TaqMan Universal PCR Master Mix (Applied Biosystems), 800 nM of each primer and 250 nM of TaqMan probe. Standard curves were generated from five dilution points for each primer pair. In the first step, amplification was started at 50 °C for 2 min and 95 °C for 10 min, followed by 40 cycles of PCRs: 95 °C for 15 s and 60 °C for 1 min. For qPCR analyses the Quant Studio3 system (ThermoFisher Scientific) was used.

The *T. aestivum* cDNA clone whv16n3n16 5- (GenBank accession number: CJ705892) was used as an internal standard for normalization of qPCR results, as it revealed the most stable expression in the tested wheat lines under experimental treatment conditions. In order to determine the best reference for qPCR, ten potential reference genes were selected: 5 of them were ‘typical’ reference genes (actin, tubulin, GAPDH, ubiquitin, and translation elongation factor) and the remaining 5 were selected from Genevestigator database using RefGenes tool. The validation of the reference genes was performed with 4 different algorithms: geNorm, NormFinder, BestKeeper, and RefFinder (data unpublished). Relative gene expression was calculated using the 2^−∆∆Ct^ method^94^. Each sample was analyzed in three full biological and three technical replicates. For determination of the specificity of qPCR reaction NTC (No Template Control) was applied for each reaction. The results were analyzed using the dedicated relative quantification software module from ThermoFisher Cloud (ThermoFisher Scientific).

### Statistical analyses

In order to determine statistical significance of the change of analyzed genes expression in comparison to respective control forms a one-way ANOVA with Dunnett’s post-hoc test was performed at 0.05 probability level based on the ∆C_T_ values. All statistical analyses were performed using Statistica 13.1 software package (Dell Inc.).

## Data Availability

The datasets generated during and/or analyzed during the current study are available from the corresponding author on reasonable request.
